# Unloading work of breathing during high-frequency oscillatory ventilation: a bench study

**DOI:** 10.1186/cc4968

**Published:** 2006-07-18

**Authors:** Marc van Heerde, Karel Roubik, Vitek Kopelent, Frans B Plötz, Dick G Markhorst

**Affiliations:** 1 Department of Pediatric Intensive Care, VU University Medical Center, Amsterdam, The Netherlands; 2 Faculty of Biomedical Engineering, Czech Technical University in Prague, Kladno, Czech Republic

## Abstract

**Introduction:**

With the 3100B high-frequency oscillatory ventilator (SensorMedics, Yorba Linda, CA, USA), patients' spontaneous breathing efforts result in a high level of imposed work of breathing (WOB). Therefore, spontaneous breathing often has to be suppressed during high-frequency oscillatory ventilation (HFOV). A demand-flow system was designed to reduce imposed WOB.

**Methods:**

An external gas flow controller (demand-flow system) accommodates the ventilator fresh gas flow during spontaneous breathing simulation. A control algorithm detects breathing effort and regulates the demand-flow valve. The effectiveness of this system has been evaluated in a bench test. The Campbell diagram and pressure time product (PTP) are used to quantify the imposed workload.

**Results:**

Using the demand-flow system, imposed WOB is considerably reduced. The demand-flow system reduces inspiratory imposed WOB by 30% to 56% and inspiratory imposed PTP by 38% to 59% compared to continuous fresh gas flow. Expiratory imposed WOB was decreased as well by 12% to 49%. In simulations of shallow to normal breathing for an adult, imposed WOB is 0.5 J l^-1 ^at maximum. Fluctuations in mean airway pressure on account of spontaneous breathing are markedly reduced.

**Conclusion:**

The use of the demand-flow system during HFOV results in a reduction of both imposed WOB and fluctuation in mean airway pressure. The level of imposed WOB was reduced to the physiological range of WOB. Potentially, this makes maintenance of spontaneous breathing during HFOV possible and easier in a clinical setting. Early initiation of HFOV seems more possible with this system and the possibility of weaning of patients directly on a high-frequency oscillatory ventilator is not excluded either.

## Introduction

Maintenance of spontaneous breathing in mechanically ventilated patients has beneficial effects. Spontaneous breathing augments ventilation perfusion matching and cardiopulmonary function, reduces sedative requirement and shortens intensive care stay [1-5]. High-frequency oscillatory ventilation (HFOV), at least in theory, achieves all goals of lung protective ventilation. It is a useful ventilatory mode for neonatal application [6,7] and is gaining interest in both paediatric and adult intensive care [8-12]. Clinical trials suggest that the use of HFOV at a lower severity threshold of acute respiratory distress syndrome improves outcome [9,13,14]. In all adult clinical trials evaluating the efficacy of HFOV, muscular paralysis was part of the study protocol [15]. In larger children and adults, spontaneous breathing during HFOV is currently advocated but usually not well tolerated because of patient discomfort. Consequently, early initiation of HFOV has to be weighed against a high level of sedation or even muscular paralysis.

In a previous bench study, we showed that spontaneous breathing of significant tidal volumes during HFOV using a SensorMedics 3100B ventilator (Yorba Linda, CA, USA) leads to a high level of imposed work of breathing (WOB) and significant swings in mean proximal airway pressure () [16]. The imposed WOB is the work added to the physiological WOB when breathing through a breathing apparatus. In a 3100B ventilator, this includes work to overcome resistance added by the endotracheal tube, the breathing circuit and the humidification device. The limited continuous fresh gas flow rate (that is to say, bias flow on a SensorMedics high-frequency oscillatory (HFO) ventilator) is the most important factor contributing to imposed WOB in the previous study. This high imposed WOB explains the patient discomfort [17]. The normal level of physiological WOB in an adult is 0.3 to 0.6 J l^-1 ^[18]. The level of imposed WOB of spontaneous breathing during HFOV exceeds this value by up to 400%.

To reduce both imposed WOB and swings in mean airway pressure on account of spontaneous breathing during HFOV, a demand-flow system has been developed. This device has to be capable of detecting patients' breathing efforts and subsequently adjust the fresh gas flow rate in order to reduce imposed WOB and pressure swings.

## Materials and methods

### Ventilator principle

**Figure 1:**
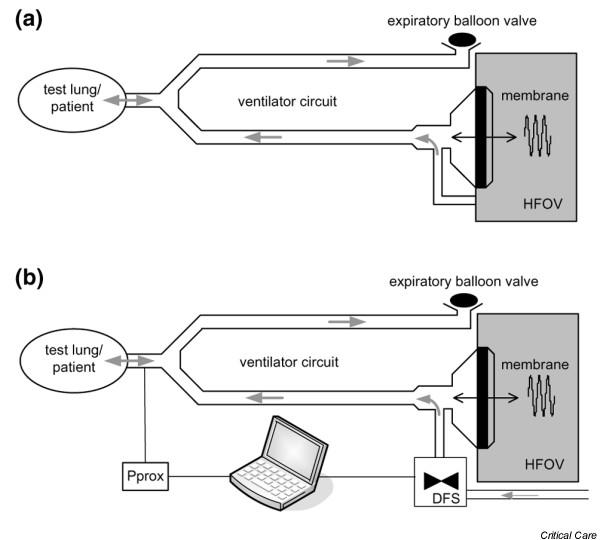
Scheme of the 3100B high-frequency oscillatory ventilator (HFOV) and the demand-flow system (DFS) connection. **(a) **The basic principle of the 3100B high-frequency oscillator. **(b) **Schematic drawing of the connection of the DFS to the 3100B oscillator. Pprox, proximal airway pressure.

The SensorMedics 3100B HFO ventilator used generates a mean airway pressure by a continuous fresh gas flow, with a maximum of 60 l minute^-1 ^(Figure 1a). This flow passes through the patient circuit and leaves the circuit via a balloon valve. This valve is inflated to a preset pressure, leading to maintenance of a stable mean proximal airway pressure. The respiratory system is brought to its mean lung volume by this pressure, enabling oxygenation. Ventilation results from pressure oscillations generated by a loudspeaker membrane at 3 to 15 Hz superimposed upon the set mean proximal airway pressure [19].

### The demand-flow system setup

The entire demand-flow system comprises two principal components: the hardware (consisting of electronic and pneumatic parts) and the control software.

**Figure 2:**
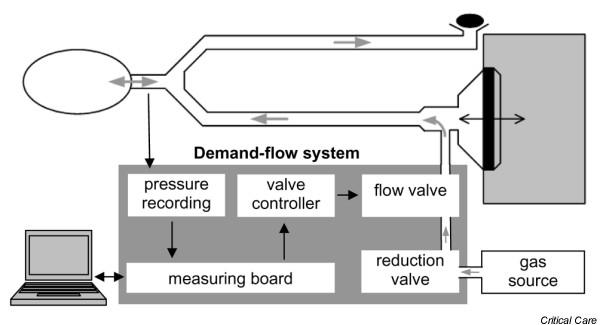
Schematic description of the demand-flow system structure.

The hardware part of the system consists of an electronically controlled mass flow valve, proximal pressure measurement sensor with a necessary electric circuit and control and communication electronics. The valve (obtained from a commercial mechanical ventilator; AVEA, Viasys Healthcare, Yorba Linda, CA, USA) provides fresh gas flow to the ventilator circuit of the 3100B HFOV ventilator (Figure 1b). A measuring board (NI_DAQ 6024E, National Instruments Corporation, Austin, TX, USA) with A/D and D/A converters assures digitization of the analogue pressure signal and its transmission into a personal computer as well as conversion of control digital data from the computer into their equivalent analogue signals suitable for control of the flow valve (Figure 2). An interface connects the measuring board with the pressure sensor and a micro proportional driver that directly actuates the flow valve. Pprox is measured by a pressure sensor (14PC03D, Honeywell, USA) at the proximal end of the endotracheal tube. This pressure signal is preprocessed in the consequent circuit and is sent to the control computer. From this signal, patient breathing effort is detected and then a control signal is sent back to the hardware part to control the proportional valve. Fresh gas flow is regulated by patient demand. During inspiration of the patient, the flow rate is increased, and, during expiration, it is decreased.

The software part of the demand-flow system is responsible for analysis of the measured Pprox and consequent control of the flow valve. The control software is developed in a Matlab^® ^environment (The Mathworks, Nattick, USA).

**Figure 3:**
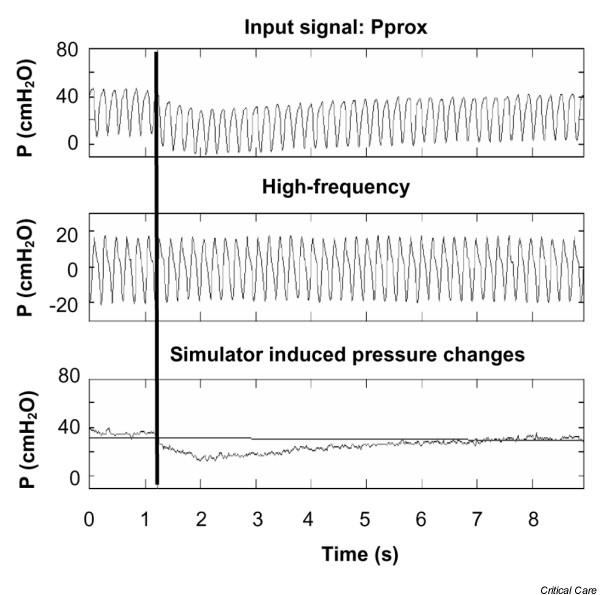
Recording during an inspiration with continuous fresh gas flow. Top panel: pressure signal sampled at airway opening (proximal airway pressure (Pprox)). Middle panel: computed high frequency component of the pressure signal, test lung influence eliminated. Bottom panel: computed test lung induced pressure changes. The vertical line denotes the start of simulated induced inspiration. The horizontal line in the bottom panel represents set mean Pprox. The curve represents fluctuation of set mean Pprox on account of breathing.

The measured pressure signal contains oscillations generated by the HFO ventilator and fluctuations generated by the patient breathing effort. To enable control of the system, the measured pressure signal (Figure 3, upper panel) is decomposed into two parts using a discreet mathematical algorithm: one component represents the ventilator pressure signal (Figure 3, middle panel) and the second part represents the patients breathing simulated with a test lung (Figure 3, lower panel). The ventilator pressure signal is a high frequency wave signal (3 to 15 Hz), in general with an asymmetrical inspiratory to expiratory time relationship. Therefore, it contains multiple higher harmonic components. The pressure signal introduced by spontaneous breathing of a patient contains lower frequency components, which allows decomposition of the measured signal into the two described parts. The patient signal is used for control of the flow valve. It modifies the delivered airflow into the ventilator circuit so that the changes in delivered airflow compensate for the pressure swings generated by the patient breathing effort. Regulation of the valve is conducted with the aim of maintaining the lowest possible deviation of the set mean Pprox. This is the way imposed WOB is reduced [18]. Decreasing amplitude of the pressure swings when the demand-flow system is in operation serves as a criterion for the control algorithm functionality. An increased airflow into the ventilator circuit during inspiration assists the patient to overcome the resistance of the ventilator circuit. It therefore reduces the breathing work required for spontaneous breathing. The system also reduces WOB during expiration because the airflow into the ventilator circuit is decreased in this phase.

### Breathing simulation

A digitally controlled test lung (high fidelity breathing simulator Active Servo Lung 5000, Ingmar Medical, Pittsburgh, PA, USA) simulated spontaneous breathing. The pressure oscillations of the HFO ventilator interfered with the spontaneous breathing modes of the test lung. For this reason, the test lung was programmed as a volume pump in the 'user-defined pressure profile' mode [20]. It generated flow patterns corresponding to spontaneous breathing flow patterns as described earlier [16]. A sinusoid flow simulated inspiration of spontaneous breathing, exponentially decelerating flow expiration.

The test lung was set to deliver tidal volumes (Vt) of approximately 340, 450 and 660 ml at a rate of 12 minute^-1 ^and a series of breaths of 420 ml at a rate of 24 minute^-1^. These settings were chosen to represent shallow and normal to deep breathing in an adult at a normal and rapid breath rate. The inspiration to expiration ratio was one to two, as in normal breathing. Each series of breaths was preceded by a breathing pause to calculate mean proximal and lung pressures. An 8.0 mm inner diameter endotracheal tube (Rüschelit, Rüsch, Kernen, Germany) connects the test lung and the HFO ventilator. Flow through the endotracheal tube was measured with a hot-wire anemometer (Florian, Acutronic Medical Systems AG, Hirzel, Switzerland). The flow signal, the pressure signal measured at the distal end of the endotracheal tube (Pett) and the Pprox signal were sampled with a sampling frequency of 100 Hz and stored for off-line analysis. Following parameters were set on the HFOV ventilator: fresh gas flow 60 l minute^-1^; mean Pprox 30 cmH_2_O; oscillatory frequency 5 Hz; and proximal pressure amplitude 80 cmH_2_O.

### Quantification of inspiratory effort

Inspiratory imposed WOB is calculated by integrating pressure measured at the distal end of the endotracheal tube (Pett) times the volume change during inspiration [21,22]:

Imposed inspiratory WOB = 

where Vt,insp stands for inhaled tidal volume. As inspiration is active and expiration usually passive, only inspiratory imposed WOB is generally considered. Application of HFOV using the SensorMedics 3100B HFO ventilator may be regarded as a super-continuous positive airway pressure system. Altering a continuous positive airway pressure device aiming at a reduction of inspiratory imposed WOB can result in an increase in expiratory imposed WOB. This may even lead to an increase in total imposed WOB [23]. Therefore, expiratory imposed work of breathing was also calculated:

Imposed expiratory WOB = 

where Vt,exp stands for exhaled tidal volume.

To enable comparison of imposed WOB in different ventilator setups, imposed WOB is often normalized, that is, related to Vt. Imposed WOB is then expressed in Joules per liter (J l^-1^). Work per liter reflects changes in pulmonary mechanics. It is influenced by changes in resistance and compliance of the respiratory system. In a SensorMedics HFO ventilator, imposed WOB is directly related to the difference in mean Pprox () level set on the ventilator and Pett; the greater the difference, the greater imposed WOB and thus patient effort.

**Figure 4:**
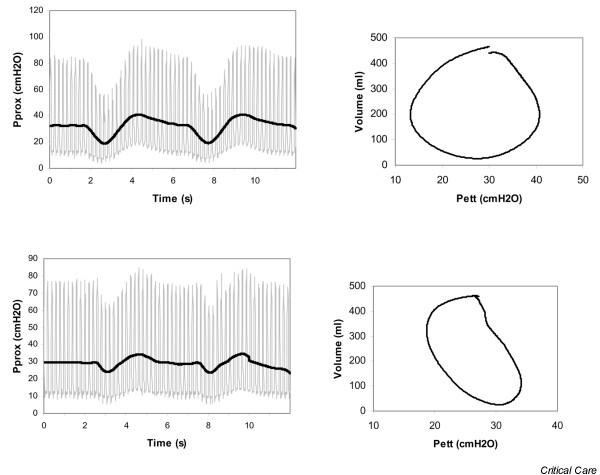
Pressure recordings of proximal airway pressure (Pprox; left panels) and modified Campbell diagrams (right panels) during simulated spontaneous breathing. Upper panels show recordings with continuous fresh gas flow; bottom panels show recordings with the demand-flow system. The left panels depict Pprox variation during two subsequent breaths. Thin lines represent unfiltered pressure signals and thick lines represent filtered Pprox. Note the reduced changes in both unfiltered and filtered signal with the demand-flow system (imposed pressure time product 17 cmH_2_O s versus 6.8 cmH_2_O s). Lines in the right panels represent mean lung pressure. Note the reduced surface area in the lower right panel. Imposed work of breathing is 1.2 J l^-1 ^without the demand-flow system versus 0.5 J l^-1 ^in the lower right panel with the demand-flow system. Pett, pressure at end of the tracheal tube.

Pprox (Figure 4, left panels), Pett and flow through the endotracheal tube were low pass filtered using a Butterworth filter, with a cut off frequency of 2 Hz. A volume signal was constructed by numerical time integration of the filtered flow signal. Subsequently, a modified Campbell diagram of each breath was plotted (Figure 4, right panels). The surface of the inspiratory part of the resulting plot represents inspiratory imposed WOB [21,22]. Since imposed WOB does not reflect isometric inspiratory effort, additionally imposed pressure-time product (PTP) was calculated from the filtered Pett signal [24]:

Imposed inspiratory PTP = 

Besides the calculation of the imposed workload, changes of Pprox on account of spontaneous breathing were measured using the filtered Pprox signal.

## Results

The demand-flow system reduced inspiratory imposed WOB by 30% to 56%, inspiratory imposed PTP by 38% to 59% and expiratory imposed WOB by 12% to 49% compared to using a maximum possible continuous fresh gas flow of 60 l minute^-1 ^(Table 1; Figure 4). Mean Pprox () changed during simulated breaths (Figure 4, left panels). With continuous fresh gas flow  fluctuated on average between -17 cmH_2_O below set  during inspiration to +11 cmH_2_O above set  during expiration (Table 1). With the demand-flow system, these fluctuations were smaller: -9 to +8 cmH_2_O. According to the oscillator manual, as a safety measure, proximal pressure alarm limits should be set 3 cmH_2_O below and above the set . With continuous fresh gas flow,  was outside these safety limits 25% of the time during simulated spontaneous breathing during inspiration and 33% during expiration. With the demand-flow system it was 11% of time during inspiration and 12% during expiration. Using the continuous fresh gas flow ventilator, alarms sounded constantly during all simulations. Using the demand-flow system this only occurred at a breathing simulation with a Vt of 420 ml 24 minute^-1^.

## Discussion

Spontaneous breathing during HFOV results in significant fluctuations of mean airway pressure. We recently described that this results in a high level of inspiratory WOB [17]. For the operator, the pressure fluctuations hamper titration of the desired set airway pressure while the pressure safety alarm limits may be exceeded frequently. This experiment demonstrates that, in a bench test, the use of the demand-flow system decreases imposed WOB significantly. It also limits breathing induced fluctuation of proximal airway pressure and time where proximal pressure exceeds safety alarm limits during breathing.

Although the demand-flow algorithm was primarily designed to reduce inspiratory WOB by increasing fresh gas flow to meet patient demand, this did not lead to increase in pressures during expiration. Expiratory imposed WOB was reduced as well. The effectiveness of the demand-flow system in decreasing the imposed WOB was less marked when breath rate increased from 12 to 24 minute^-1^. In paediatric patients, but also in some adults, with severe acute respiratory distress syndrome, high breathing rates at small tidal volumes are clinically observed. The effectiveness of the demand-flow system needs, therefore, to be tested *in vivo*.

The optimal workload for critically ill patients is unclear. It depends on energy and muscular reserve. Research focuses mainly on WOB in the weaning phase [25,26]. A WOB level in the physiological range, approximately 0.5 J l^-1 ^in adults, seems to correspond with an optimal workload. Full unloading, for instance reducing the WOB to zero, induces loss of respiratory muscles. Excessive respiratory muscle loading may cause muscle fatigue and weaning failure [26]. This workload of 0.5 J l^-1 ^seems to be optimal not only during weaning, but also in the acute phase of respiratory failure [3,27]. Compared to the WOB of a healthy adult (0.3 to 0.6 J l^-1^), imposed WOB is high if spontaneous breathing is simulated during HFOV using continuous fresh gas flow. Using the demand-flow system, imposed WOB is considerably reduced. In simulations of shallow to normal breathing for an adult, imposed WOB was 0.5 J l^-1 ^at maximum.

The SensorMedics 3100A HFO ventilator was originally designed for neonatal application. The 3100B ventilator was thereafter designed for oscillating patients weighing more than 35 kg. Although small neonatal patients can breathe comfortably on their HFOV circuit, paralysis has been felt necessary in most larger patients in whom spontaneous breathing imposes a high level of WOB and triggers numerous alarms, with resultant loss of the benefits of maintaining some element of spontaneous respiration during ventilator support. This is a significant disadvantage that needs rectification [7,16]. The demand-flow system seems capable of achieving this.

### Limitations of the study

In this *in vivo *study we aimed to choose realistic test conditions. The limitations of the bench test model were discussed in the previous study [17]. In addition, patient ventilator interaction cannot completely be simulated in a bench test. Whether the demand-flow system is capable of providing comfortable ventilation synchrony, for instance, needs to be tested *in vivo*. In future studies, we will investigate this effect and the clinical applicability of the device in spontaneously breathing subjects.

## Conclusion

A novel demand-flow system has been designed that is capable of automatic regulation of HFO ventilator fresh gas flow adjusted to patient need during simulated spontaneous breathing. This results in a considerable reduction of imposed WOB and reduction of swings in mean Pprox. The amount of reduction in imposed WOB is promising. Potentially, this may lead to a reduced use of sedatives and muscular paralysis in larger patients during HFOV. Early initiation of HFOV seems more possible with this system and the possibility of weaning of patients directly on a HFO ventilator is not excluded either.

## Key messages

• 	A demand-flow system was developed to reduce imposed WOB during HFOV.

• 	This system is able to reduce imposed WOB to the physiological range of 0.5 J l^-1^.

• 	Fluctuations in set mean Pprox are effectively reduced so that alarm limits are not exceeded.

• 	This potentially leads to better acceptance of spontaneous breathing during HFOV.

• 	Early initiation of HFOV seems more possible with this system and the possibility of weaning of patients directly on a HFO ventilator is not excluded either.

## Abbreviations

HFO = high-frequency oscillatory; HFOV = high-frequency oscillatory ventilation; Pett = pressure at end of the tracheal tube; Pprox = proximal airway pressure; PTP = pressure time product; Vt = tidal volume; WOB = work of breathing.

## Competing interests

The authors declare that they have no competing interests.

## Authors' contributions

MvH designed the study, conducted the bench study, analysed the results and drafted the manuscript. KR and VK developed the demand-flow system, and participated in the bench study and analysis of the results. FBP participated in interpreting the results. DGM assisted in designing the study, and participated in interpreting the results and drafting the manuscript. All authors read and approved the final manuscript.

## Figures and Tables

**Table 1:** Summary of test results with continuous flow and the demand-flow system

Simulated breaths		Δ (cmH_2_O)				WOBi (J l^-1^)		PTPi (cmH_2_O s)		WOBe (J l^-1^)	
Vt (ml)	Rate (min^-1^)	CF		DFS		CF	DFS	CF	DFS	CF	DFS

330	12	-10	+6	-4	+4	0.85	0.37	12	5.1	0.51	0.26
450	12	-13	+9	-6	+5	1.2	0.50	17	6.8	0.69	0.38
660	12	-20	+14	-9	+7	1.8	0.78	26	11	1.1	0.61
420	24	-24	+16	-15	+17	2.2	1.5	16	10	1.4	1.2

Simulated breaths: Vt, tidal volume; rate, breathing frequency. Δ; deviation of mean proximal airway pressure from set  during inspiration (negative value) and expiration (positive value). WOBi; inspiratory imposed work of breathing. PTPi; inspiratory imposed pressure time product. WOBe; expiratory imposed work of breathing. CF, continuous flow. DFS, demand-flow system.

## References

[B1] Cereda M, Foti G, Marcora B, Gili M, Giacomini M, Sparacino ME, Pesenti A (2000). Pressure support ventilation in patients with acute lung injury. Crit Care Med.

[B2] Putensen C, Mutz NJ, Putensen-Himmer G, Zinserling J (1999). Spontaneous breathing during ventilatory support improves ventilation-perfusion distributions in patients with acute respiratory distress syndrome. Am J Respir Crit Care Med.

[B3] Putensen C, Muders T, Varelmann D, Wrigge H (2006). The impact of spontaneous breathing during mechanical ventilation. Curr Opin Crit Care.

[B4] Sydow M, Burchardi H, Ephraim E, Zielmann S, Crozier TA (1994). Long-term effects of two different ventilatory modes on oxygenation in acute lung injury. Comparison of airway pressure release ventilation and volume-controlled inverse ratio ventilation. Am J Respir Crit Care Med.

[B5] Wrigge H, Zinserling J, Neumann P, Muders T, Magnusson A, Putensen C, Hedenstierna G (2005). Spontaneous breathing with airway pressure release ventilation favors ventilation in dependent lung regions and counters cyclic alveolar collapse in oleic-acid-induced lung injury: a randomized controlled computed tomography trial. Crit Care.

[B6] Bollen CW, Uiterwaal CS, van Vught AJ (2003). Cumulative metaanalysis of high-frequency versus conventional ventilation in premature neonates. Am J Respir Crit Care Med.

[B7] Froese AB, Kinsella JP (2005). High-frequency oscillatory ventilation: lessons from the neonatal/pediatric experience. Crit Care Med.

[B8] Bollen CW, van Well GT, Sherry T, Beale RJ, Shah S, Findlay G, Monchi M, Chiche JD, Weiler N, Uiterwaal CS, van Vught AJ (2005). High frequency oscillatory ventilation compared with conventional mechanical ventilation in adult respiratory distress syndrome: a randomized controlled trial [ISRCTN24242669]. Crit Care.

[B9] Derdak S, Mehta S, Stewart TE, Smith T, Rogers M, Buchman TG, Buchman TG, Carlin B, Lowson S, Granton J (2002). High-frequency oscillatory ventilation for acute respiratory distress syndrome in adults: a randomized, controlled trial. Am J Respir Crit Care Med.

[B10] Singh JM, Ferguson ND (2005). Is it time to increase the frequency of use of high-frequency oscillatory ventilation?. Crit Care.

[B11] Slee-Wijffels FY, van der Vaart KR, Twisk JW, Markhorst DG, Plotz FB (2005). High-frequency oscillatory ventilation in children: a single-center experience of 53 cases. Crit Care.

[B12] Pachl J, Roubik K, Waldauf P, Fric M, Zabrodsky V (2006). Normocapnic high-frequency oscillatory ventilation affects differently extrapulmonary and pulmonary forms of acute respiratory distress syndrome in adults. Physiol Res.

[B13] David M, Weiler N, Heinrichs W, Neumann M, Joost T, Markstaller K, Eberle B (2003). High-frequency oscillatory ventilation in adult acute respiratory distress syndrome. Intensive Care Med.

[B14] Mehta S, Granton J, MacDonald RJ, Bowman D, Matte-Martyn A, Bachman T, Smith T, Stewart TE (2004). High-frequency oscillatory ventilation in adults: the Toronto experience. Chest.

[B15] Derdak S (2003). High-frequency oscillatory ventilation for acute respiratory distress syndrome in adult patients. Crit Care Med.

[B16] van Heerde M, van Genderingen HR, Leenhoven T, Roubik K, Plotz FB, Markhorst DG (2006). Imposed work of breathing during high-frequency oscillatory ventilation: a bench study. Crit Care.

[B17] Sessler CN (2005). Sedation, analgesia, and neuromuscular blockade for high-frequency oscillatory ventilation. Crit Care Med.

[B18] Banner MJ, Jaeger MJ, Kirby RR (1994). Components of the work of breathing and implications for monitoring ventilator-dependent patients. Crit Care Med.

[B19] Pillow JJ (2005). High-frequency oscillatory ventilation: mechanisms of gas exchange and lung mechanics. Crit Care Med.

[B20] IngMar Medical (2003). Operator Instructions ASL 5000.

[B21] Campbell EJ (1958). The Respiratory Muscles and the Mechanisms of Breathing.

[B22] Agostini E, Campbell EJ, Freedman S, Campbell EJ, Agostini E, Newsom Davis J (1970). Energetics. The Respiratory Muscles.

[B23] Banner MJ, Downs JB, Kirby RR, Smith RA, Boysen PG, Samsun Lampotang ME (1988). Effects of expiratory flow resistance on inspiratory work of breathing. Chest.

[B24] French CJ (1999). Work of breathing measurement in the critically ill patient. Anaesth Intensive Care.

[B25] Girault C, Breton L, Richard JC, Tamion F, Vandelet P, Aboab J, Leroy J, Bonmarchand G (2003). Mechanical effects of airway humidification devices in difficult to wean patients. Crit Care Med.

[B26] MacIntyre NR (2005). Respiratory mechanics in the patient who is weaning from the ventilator. Respir Care.

[B27] MacIntyre NR, Cook DJ, Ely EW, Epstein SK, Fink JB, Heffner JE, Hess D, Hubmayer RD, Scheinhorn DJ (2001). Evidence-based guidelines for weaning and discontinuing ventilatory support: a collective task force facilitated by the American College of Chest Physicians; the American Association for Respiratory Care; and the American College of Critical Care Medicine. Chest.

